# Considerations on the role of environmental toxins in idiopathic Parkinson’s disease pathophysiology

**DOI:** 10.1186/2047-9158-3-10

**Published:** 2014-05-09

**Authors:** Francisco Pan-Montojo, Heinz Reichmann

**Affiliations:** 1 Neurologische Klinik, Klinikum der Ludwig-Maximilians-Universität München, Marchioninistr. 15, 81377 Munich, Germany; 2 Munich Cluster for Systems Neurology (SyNergy), Adolf-Butenandt-Institut Ludwig-Maximilians-Universität München, Schillerstr. 44, 80336 Munich, Germany; 3 Klinik und Poliklinik für Neurologie, Carl Gustav Carus University Hospital, TU-Dresden, Fetscherstr. 74, 01307 Dresden, Germany

**Keywords:** Idiopathic Parkinson’s disease, Environmental toxins, Gene-environment interactions, Braak’s staging and pathology progression

## Abstract

Neurodegenerative diseases are characterized by a progressive dysfunction of the nervous system. Often associated with atrophy of the affected central or peripheral nervous structures, they include diseases such as Parkinson’s Disease (PD), Alzheimer’s Disease and other dementias, Genetic Brain Disorders, Amyotrophic Lateral Sclerosis (ALS or Lou Gehrig’s Disease), Huntington’s Disease, Prion Diseases, and others. The prevalence of neurodegenerative diseases has increased over the last years. This has had a major impact both on patients and their families and has exponentially increased the medical bill by hundreds of billions of Euros. Therefore, understanding the role of environmental and genetic factors in the pathogenesis of PD is crucial to develop preventive strategies. While some authors believe that PD is mainly genetic and that the aging of the society is the principal cause for this increase, different studies suggest that PD may be due to an increased exposure to environmental toxins. In this article we review epidemiological, sociological and experimental studies to determine which hypothesis is more plausible. Our conclusion is that, at least in idiopathic PD (iPD), the exposure to toxic environmental substances could play an important role in its aetiology.

## Introduction

PD affects around 1% of the general population a rate that increases with age reaching up to 5% of the 80 years old population [1]. Its annual incidence is between 16 and 19 per 100,000 per year [2]. It affects all races equally, with slight male predomination. In Europe PD prevalence is 1,6/100 inhabitants, with a wide variability between different countries [3]. Although multiple genetic forms of the disease have been observed [4], they account for less than 10-15% of the total cases and their importance vary between different regions [5]. If iatrogenic and vascular parkinsonisms are included together with complex degenerative diseases and atypical parkinsonisms, the total prevalence increases to 2,3/100. During the twentieth century, the general incidence of PD increased 1.63 times. This increase occurred mostly during the first half until 1980 and affected developed countries [6,7]. Similarly, other neurodegenerative diseases have dramatically increased in some regions and decreased in others [8]. Interestingly, this increase is most evident in developed countries and within these countries in those regions using agrochemical compounds [9-11]. Some studies claim that such an increase is due to the aging of the population, associated with mitochondrial dysfunction and a reduced protective response to oxidative stress, other studies point to the interaction between environmental toxins and diverse genetic backgrounds as the main triggers of PD. In this work, we show evidence supporting these two different hypotheses in relation with the most recent findings on PD pathophysiology with special emphasis on iPD.

### A historic perspective on Parkinson’s disease and its relation with toxic and protective environmental substances

The English physician James Parkinson in his work “An essay on the Shaking Palsy” described PD for the first time in 1817. This correlates well with the beginning of the industrial and chemical revolution in Europe during the late 18^th^ century and the 19^th^ century. To our knowledge, only the Ayuverda (the medical system practiced in India around 5000 years before Christ) and the first Chinese manuscript on medicine, Nei Ping, written 2500 years ago, describe some of the symptoms observed in PD and potential treatments [12,13]. Apart from this reference to PD-related symptoms, no other physician in any of the occidental countries had previously described the complex amalgam of symptoms typical of this disorder. This suggests that either these different symptoms had always been misdiagnosed as separate entities throughout history, not recognized as part of a syndrome, or the prevalence of PD until the beginning of the 19^th^ century had been extremely low. While possible, the first hypothesis seems quite improbable to us. Many PD symptoms are quite striking and would have been described and published before Parkinson’s assay. Therefore, we believe that a dramatic increase of PD cases occurred in parallel to the industrial revolution. In this case, the question is, why?

Some authors have related the increase of PD incidence to the aging of the population [14,15]. Aging has been associated to the impairment of the antioxidant body system and mitochondrial function [16]. It is known that age-related non-genetic PD appears between the 5^th^ and 6^th^ decade of life. Logically, an increase in life expectancy would lead to an increased incidence and prevalence of PD. However, in the last 1000 years life expectancy at 15 years of age (i.e. discounting birth and child mortality) has always been above these values [17,18]. Interestingly, a general increase in PD incidence was observed during the first half of the twentieth century from 11,4/100.000 inhabitants between 1935 and 1944 to 18,2/100.000 inhabitants in both young and old populations between 1967 and 1979 [6,7]. Since then it has remained more or less constant due to a compensation effect. There has been a decrease in the incidence rate in the population under 69 years of age and an increase in the population over 70 years [6]. Altogether, these data suggests that the increase in PD incidence cannot be due to the aging of the population alone.

Accordingly, the appearance of iPD has been related to industrial and rural environments due to the higher exposure to environmental toxins [10,19]. Natural pesticides have been used since more than 5000 thousand years. The first known pesticide was elemental sulfur dusting used about 4,500 years ago. Also, the Rig Veda, which is about 4,000 years old, mentions the use of poisonous plants for pest control [20]. However, it was not until the 15th century that toxic chemicals such as arsenic, mercury, and lead were applied to crops to kill pests. Later, nicotine sulfate was extracted from tobacco leaves for use as an insecticide in the 17th century. Remarkably, only in the 19th century two pesticides related to PD, pyrethrum, derived from chrysanthemums, and rotenone, which is derived from the roots of tropical vegetables, started to be used [21].

Other more recent epidemiological studies have broadened the link between PD and other environmental factors including drinking well water, rural living, farming, diet and exposure to agricultural chemicals [22-24]. The environmental contribution to PD’s pathophysiology has been also analysed. Farm and industrial compounds seem to increase the risk of Parkinsonism [11,22,25-28]. Farm activity is associated with agriculture and exposure to pesticides [24]. Organochlored pesticides were identified as a risk factor in a German case-control study [27] and another study with similar conclusions was conducted with dithiocarbamates [28]. Levels of organochlorines have been found to be elevated in the brains of persons with iPD [29]. A study of French elderly individuals found an association between past occupational exposures to pesticides, low cognitive performance, and increased risk of developing Alzheimer’s disease or iPD [9]. In a more recent epidemiological study, Tanner and colleagues have tried to identify a common characteristic of those pesticides that present a higher correlation to the appearance of PD [30]. They conclude that pesticides inhibiting the mitochondrial Complex I and increasing oxidative stress are more prone to induce iPD upon exposure. Together with pesticides and herbicides it has been observed that some xenobiotics like annonacin induce Parkinsonian symptoms in humans and a loss of nigrostriatal neurons in animals [31].

iPD has also been linked to the exposure to different metals and industrial compounds. Many studies performed in the 90’s identified manganese, lead, copper, iron, zinc, aluminium or amalgam (reviewed in [24]). Higher incidence of iPD has been reported in manganese miners [32]. It was shown that manganese, a component of various pesticides, also reproduces parkinsonian symptoms after long and chronic exposures (between 6 months and 16 years). Also, concern was raised that widespread introduction of the manganese-containing fuel additive methylcyclopentadienyl manganese tricarbonyl to the U.S. gasoline supply may increase population exposure to manganese and thus increase risk of parkinsonism in sensitive populations [33]. A more recent study has also shown a positive correlation between ß-Hexachlorocyclohexane blood levels and iPD [34].

The positive relation between exposure to environmental toxins and neurodegenerative diseases is not limited to PD. For example, the Chamorros population of Guam and Rota in the western Pacific has an unusually high prevalence of motor neuron disease, a syndrome that includes amyotrophic lateral sclerosis, parkinsonism, and progressive dementia. It was proposed that this syndrome of parkinsonian dementia is related to the consumption of flour made from cycad seeds [35] or to inhalation of pollen from cycad plants [36]. Later findings suggest that a neurotoxic non-protein amino acid, beta-methylamino-L-alanine synthesized by a symbiotic cyanobacterium highly present on cycad seeds and pollen is actually responsible for this effect [37].

Remarkably, in some populations there has been a decreasing prevalence of certain types of neurodegenerative diseases that coincide with the disappearance of an environmental factor unique to these populations [38,39].

Together with exposure to environmental toxins, PD has been related to head trauma [40-43], inflammation [44,45] and constipation [46]. While head trauma and inflammation are associated with vascular and post-encephalitic parkinsonisms with a lower progression rate (around 50%) to higher nervous structures [47], constipation is associated with iPD and patients present pathology progression. We hypothesize that, in these cases, a decreased frequency of bowel movements might increase the time that environmental toxins remain in the intestine and can interact with the organism.

As a counterbalance, certain factors have been associated with a decreased risk of developing PD. Cigarette smoking, coffee drinking and high levels of urate in blood have been negatively correlated with the appearance of PD [48-50]. The mechanisms underlying such protection are still unclear.

Therefore, the question is, can the interaction with environmental toxins explain the distribution and appearance pattern of PD clinical symptoms and pathology in non-genetic cases?

### Clinicopathological correlation in Parkinson’s disease

PD is traditionally defined by a series of clinical symptoms. These are predominantly motor disorders that give rise to the rigid-akinetic syndrome. PD is the main aetiology of rigid-akinetic syndromes. Nevertheless, non-motor symptoms are widely distributed in PD patients.

**Figure 1:**
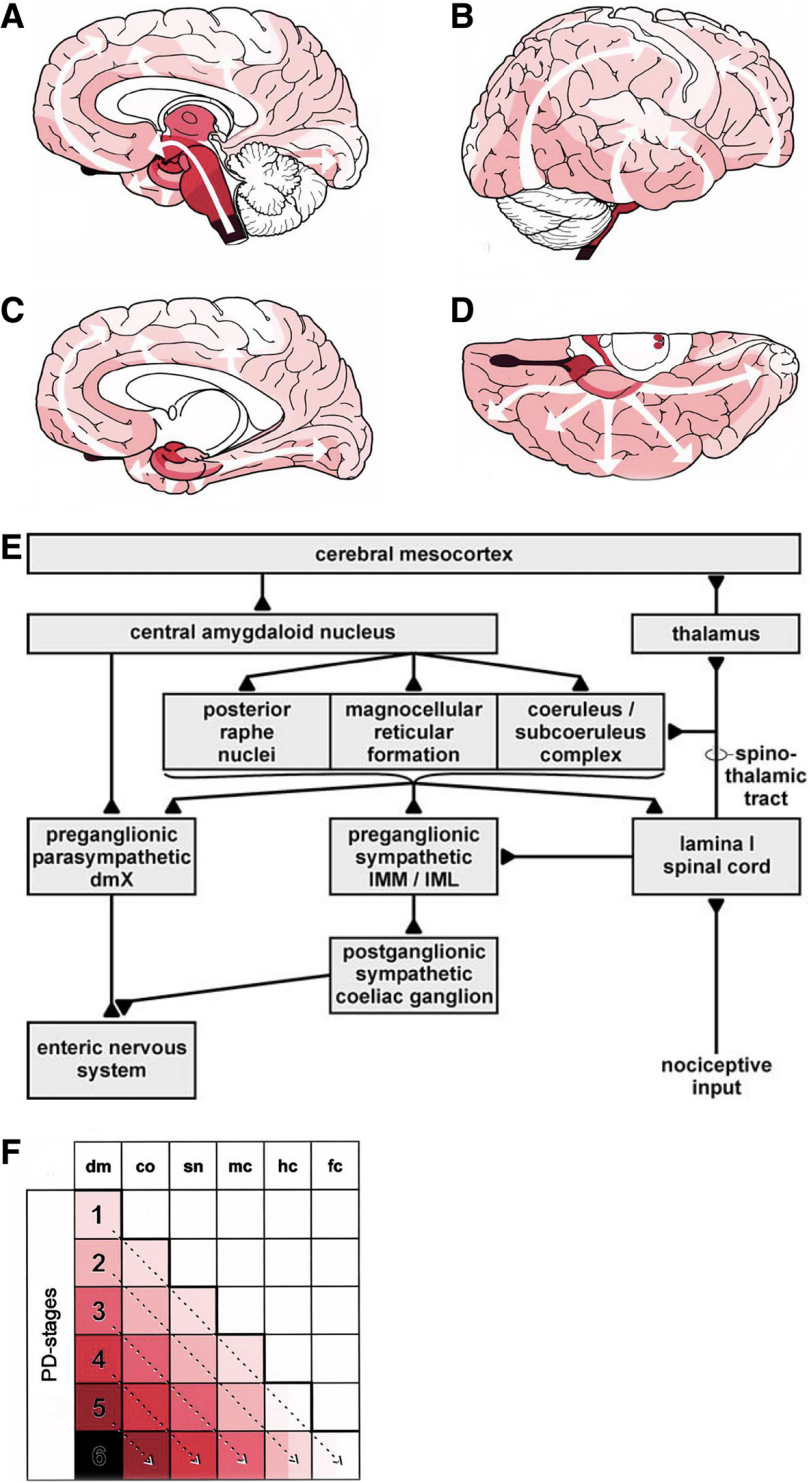
**Braak**’**s staging of Parkinson**’**s disease pathology progression. (A**-**D)** Illustrations showing the intracerebral progression of PD pathology. **(E)** Schematics of the pathology progression from the ENS. **(F)** Correlation between PD staging and the appearance of the pathology in different intracerebral structures. Modified from Braak et al. [53].

The pathophysiological implications of PD-related alterations depend on the structures affected at each stage. The main pathological findings in PD are the presence of Lewy Bodies (LB) or neurites (LN) and the loss of chatecholaminergic neurons in the locus coeruleus and the *substantia nigra* (SN). The classical appearance of the LB in pigmented neurons with hematoxylin/eosin staining is that of one or more eosinophilic spherical body with a dense core surrounded by a halo [51]. Lewy bodies are intracytoplasmatic protein accumulations consisting mainly in alpha-synuclein [52]. Based on their findings and that from others at that time, Braak and colleagues suggested that, in iPD, the pathology follows a specific progression pattern appearing first in the olfactory bulb (OB) and the dorsal motor nucleus of the vagus (DMV) [53,54], giving rise to Braak’s pathological staging of PD (Figure 1). Interestingly, similar studies had shown that LB and LN could be observed in the peripheral nervous system (PNS) including the enteric nervous system (ENS) [55,56], the sympathetic ganglia [55], the submandibular glands [57] and the cardiac plexus [58] among others. Further pathological studies observed that other central nervous system sites (i.e. the intermediolateral column and the lamina I of the posterior horn from the spinal cord) also show LBs and LNs in the initial stages of the disease. The order of appearance of the pathology throughout all these sites made Braak’s group incorporate this to their staging and established a new pathological staging including the sympathetic and parasympathetic systems and the ENS. The spatio-temporal pattern of this staging suggests that iPD-related alterations appear first in the ENS and the OB progressing into and throughout the CNS. This progression correlates well with the evolution of the clinical symptoms observed in iPD patients.

Corresponding to structural alterations, there are motor and non-motor symptoms in iPD. The onset of motor features correlates with the loss of dopamine input to the posterior putamen, corresponding to the motor region of the striatum. The main classical features of PD are therefore mainly related to the dysfunction of the motor circuit. As the disease progresses and the loss of dopaminergic neurons increases, the dopaminergic input to other areas of the striatum and the cortex (prefrontal and limbic circuits) decreases, giving rise to clinical symptoms characteristic of the dysfunction of higher cerebral structures.

Less frequent motor-symptoms that can be found in PD patients are hypophonia, taquiphemia, drooling, dysphagia, fatigue (can also be considered a non-motor symptom), hypomimia, impaired fine motor dexterity and motor coordination, impaired gross motor coordination, akathisia and palilalia. Most of these less frequent motor-symptoms seem to be expressions of the same pathophysiological alterations described above for the main motor symptoms.

Non-motor symptoms are believed to derive from the degeneration of non-dopaminergic (i.e. noradrenergic, serotoninergic and cholinergic) cellular systems. This can be applied to dementia, depression, sleep and vegetative disorders. It is known that almost 90% of PD patients experience non-motor manifestations during the course of disease [59] and are a significant economic burden [60] (with a substantial impact on the health-related quality of life). Further clinical studies show that a loss of olfaction, rapid eye movement (REM) sleep behaviour disorders and constipation anticipate motor-problems [61]. All this suggests that PD is a progressive disease, which might originate at the olfactory bulb and the ENS. Recent follow-up studies have successfully investigated the use of hyposmia/anosmia or REM sleep behaviour as early signs for PD and to test pre-motor symptoms of the disease [61-64]. Interestingly, the ENS and the OB are the nervous system structures most exposed to environmental toxins. Therefore, it seems possible that the effect of these substances on these structures could trigger the appearance and progression of the disease.

### Genetic forms of Parkinson’s disease

Despite all this body of evidence, the relatively low incidence of PD suggests that the individual genetic background plays an important role in the pathogenesis of the disease. What are the genetic alterations that predispose to the development of PD and why?

Already a century ago it was noticed that PD patients had affected relatives [65]. The role of genetic inheritance in PD has been increasingly important during the past couple of decades because of different studies. Despite the complexity of achieving good quality epidemiologic studies due to diagnostic difficulties, different studies have confirmed that PD is more common between family members [66-70]. Generally, the risk of having the disease among relatives is 2 to 3 times greater than in the general population [4]. Also, studies on homozygotic twins have shown that although there is no significant concordance in late onset disease cases [71], it becomes significant in early onset cases. Therefore, one could say that early PD is usually genetically determined.

In the last decades, there has been an increase in the number of PD family based studies [4,67,72-82]. Most of these show an autosomic pattern, either dominant or recessive. These studies have been able to identify some genetic mutations and chromosomal loci responsible for familiar PD. The most studied and known mutations are annotated in Table 1. Interestingly, a recent meta-analysis on more than 800 published genetic associations studies revealed eleven loci showing genome-wide significant association with disease risk: *BST1*, *CCDC62*/*HIP1R*, *DGKQ*/*GAK*, *GBA*, *LRRK2*, *MAPT*, *MCCC1*/*LAMP3*, *PARK16*, *SNCA*, *STK39*, and *SYT11*/*RAB25*. In addition, they identified novel evidence for genome-wide significant association with a polymorphism in *ITGA8*[83]. The list of hits is available under http://www.pdgene.org.

**Table 1:** Known genetic mutations in PD

**Locus**	**Chromosome**’**Location**	**Gene**	**Inheritance**	**Typical pheno**-**type**	**Reference**
PARK1 & PARK4	4q21-q23	α-synuclein	AD	Earlier onset, features of DLB’common	[72,73]
PARK2	6q25.2-q27	parkin	usually AR	Earlier onset with slow progression	[74]
PARK3	2p13	unknown	AD, IP	Classic PD,’sometimes de-mentia	[4]
PARK5	4p14	UCH-L1	AD	Classic PD	[75]
PARK6	1p35-p36	PINK1	AR	Earlier onset with’slow progression	[76]
PARK7	1p36	DJ-1	AR	Earlier onset with’slow progression	[67]
PARK8	12p11.2-q13.1	LRRK2	AD	Classic PD	[77]
PARK10	1p32	unknown	Unclear	Classic PD	[78]
PARK11	2q36-q37	unknown	Unclear	Classic PD	[79]
NA	5q23.1-q23.3	Synphilin1	Unclear	Classic PD	[80]
NA	2q22-q23	NR4A2	Unclear	Classic PD	[81]

*Abbreviations*: *NA* not assigned, *AD* autosomic dominant, *AR* autosomic recesive, *IP* incomplete penetrante, *DLB* Lewy Bodies Demence. Modified from [84].

Animal genetic models of the disease have been important to better understand the mechanisms underlying PD pathophysiology. Different animal models mimicking the genetic alterations observed in PD patients have been developed in organisms such as mice, worms, flies or zebrafish [85-88]. These include the knock-out, over-expression or expression of mutated forms of *PARK*-*1* (i.e. alpha-synuclein or its *A53T*, *A30P*, and *E46K* mutations) or the knock-down of *DJ*-*1*, *PINK* or *LRRK2* (*G2019S* and *R1441C*/*G* mutants) [85,89] among others. However, most of these models failed to reproduce overt nigrostriatal dopaminergic loss having wider effects throughout the CNS. In some cases, these genetic alterations even had a neuroprotective effect (e.g. over-expression of wild-type alpha-synuclein) [90,91]. Moreover, genetic mutations in PD account for less than 10% of the patients and cannot explain many of the clinical and pathological signs observed in idiopathic PD patients. Thus, it seems that environmental toxins might be playing a more important role than previously thought.

### Evidence obtained using toxic models of PD

Based on the above-mentioned observations, numerous groups have tested the effect of environmental toxins on animal and in vitro cellular models. The most common models used up to date are:

#### Animal models

These have been extensively reviewed in the literature [92-94] and we will briefly describe some of them here.

### 1-methyl-4-phenyl-1,2,3,6-tetrahydropyridine (MPTP)

MPTP is a non-toxic compound that may be accidentally produced during the manufacture of MPPP, a synthetic opioid drug. In the 80ies, several cases of Parkinson after the accidental ingestion of MPTP were described [95,96]. When ingested, it is metabolized into the toxic cation 1-methyl-4-phenylpyridinium (MPP^+^) by the enzyme MAO-B of glial cells. MPP + is a potent mitochondrial Complex I inhibitor that primarily kills dopaminergic neurons [97]. Models based on this substance have been used to understand the effect of mitochondrial inhibition, to test different neuroprotective strategies or to observe the effect of dopamine absence in different brain functions and areas [98,99]. As PD model, it presents two main problems. First, MPTP induces an acute or subacute neurodegeneration, different to the chronic PD process and second, there is no LB formation [100] and no pathology progression has been observed so far.

### 6-hydroxydopamine (6-OHDA)

6-OHDA Treatment led to the first known animal model of PD [101,102]. 6-OHDA is injected into the medial forebrain bundle of rat brain (destroying dopamine neurons in the substantia nigra pars compacta with the subsequent loss of dopamine nerve terminals in the striatum [101,102]. The unilaterally lessoned animals circle toward their lesioned side. This is driven by the asymmetric release of dopamine from the intact side of striatum [101]. 6-OHDA generates quinones inside the neurons. These quinones generate free radicals that inactivate biological macromolecules. It is necessary to inject 6-OHDA directly in the central nervous system (CNS), as it is not able to cross the brain-blood barrier. As in the case of MPTP, this model does not produce the characteristic LB nor does it show pathology progression.

### Paraquat

Paraquat is a herbicide that induces dopaminergic degeneration and LB formation in the SN of mice [103]. Its parenteral administration produces its effect by inducing superoxide radical formation. However, it is not known whether this effect is local on SN neurons or also other cell types might be affected. Moreover, pathology progression has not been reported.

### Rotenone

Rotenone is a naturally occurring pesticide derived from the roots of certain plant species that acts through mitochondrial Complex I inhibition. Rotenone has been used through non-natural ways of administration such as direct nigrostriatal infusion and systemic intraperitoneal or intravenous administration to generate toxic models of PD in rats and mice [104-107]. To achieve a more natural way of exposure to environmental toxins, two groups have used orally administered rotenone to generate PD-like pathology and symptoms in mice [108,109].

Systemic chronic administration (more the 5 weeks) of rotenone induces specific dopaminergic neuron degeneration with the formation of LB-like alpha-synuclein inclusions [104]. Moreover, high doses of rotenone lead to a striatal degeneration without SN impairment [110], showing the same degeneration pattern as in manganese and carbon monoxide exposure in primates and humans. However, systemic administration of this substance mimics a multisytemic degeneration rather than the degeneration pattern observed in PD patients [111].

Oral administration of rotenone induces different effects depending on the concentration at which it is administered. Inden and colleagues have shown that high doses (>5 mg/kg) of orally administered rotenone affect SN dopaminergic neurons one month after administration [108]. In a later study, we showed that at these high doses, dopaminergic degeneration was due to the presence of rotenone in the systemic blood [109]. Interestingly, in this same study we showed that long-time exposure to low doses of orally administered rotenone induced the appearance of PD-like pathology and its progression from the ENS into the CNS accompanied by dopaminergic loss in the SN. We did not observe systemic Complex I inhibition or the presence or rotenone in the blood or the brain. Thus, suggesting that, as the ENS and the OB are the nervous structures most exposed to environmental toxins, environmental toxins acting locally on these nervous structures trigger the appearance of PD-like pathology and its progression into the CNS through synaptically connected structures. Indeed, in a recent study, we have shown that the resection of the vagal or sympathetic nerves (connecting the ENS to the CNS) interrupts the progression of the pathology to the previously connected structures [112]. Interestingly, the co-treatment with a compound inhibiting alpha-synuclein aggregation also reduced the effect of oral administered rotenone [113].

#### In vitro cellular models

In vitro systems are very efficient screening tools for detecting potential neurotoxic compounds among the multitude of chemicals to which humans are exposed. They also offer many opportunities to investigate the cellular and molecular effects of toxins. Studies performed in primary neuronal cultures and both PC12 and SH-SY5Y cell lines have been used to test different compounds potentially involved in neurodegeneration. For example, aluminium, copper and iron, as well as several pesticides were shown to trigger structural transformation and fibrillation of alpha-synuclein [114,115]. A dithiocarbamate fungicide altered the function of the ubiquitin-proteasome system by inhibition of the ubiquitin E1 ligase [116] and different reports show that xenobiotics induce oxidative stress. Evidence for oxidative stress was also found in vitro in primary cultures of cerebellar granule neurons after exposure to numerous pesticides and insecticides [117] and [118], in PC12 cells after exposure to trimethyltin [119], in primary cultures of mesencephalic neurons after exposure to ethylene-bis-dithiocarbamate fungicide [120], and in midbrain slice cultures after exposure to the pesticide rotenone [106].

In vitro, environmental compounds have also been shown to induce glial reactivity, a crucial step of the brain inflammatory pathway. After subchronic exposure to mercury compounds, microgliosis and astrogliosis were found in aggregating brain cell cultures, without any sign of neuronal damage [121,122].

### Is there a common toxic mechanism in all these models that leads to neurodegeneration?

One of the common effects exerted by most of these noxious compounds tested above is the inhibition of mitochondrial NADH CoQ reductase, also known as Complex I, and the production of free radicals, thereby also increasing cellular oxidative stress. The first association between a mitochondrial alteration and PD was made in 1989. Two different groups showed a defect in Complex I activity from SN neurons in PD patients [123]. Later studies have shown that there is an approximately 35% defect in the mitochondrial complex I activity [124]. This deficiency is also present in platelets from PD patients [125]. As mentioned above, a study published in 2011 underlines the importance of Complex I inhibition and oxidative stress in PD pathophysiology in patients. In an epidemiological study, Tanner and colleagues observed in 110 PD cases and 358 controls that PD was strongly associated with the use of a group of pesticides that inhibit mitochondrial complex I, including rotenone, and with the use of a group of pesticides that cause oxidative stress, including paraquat [30].

**Figure 2:**
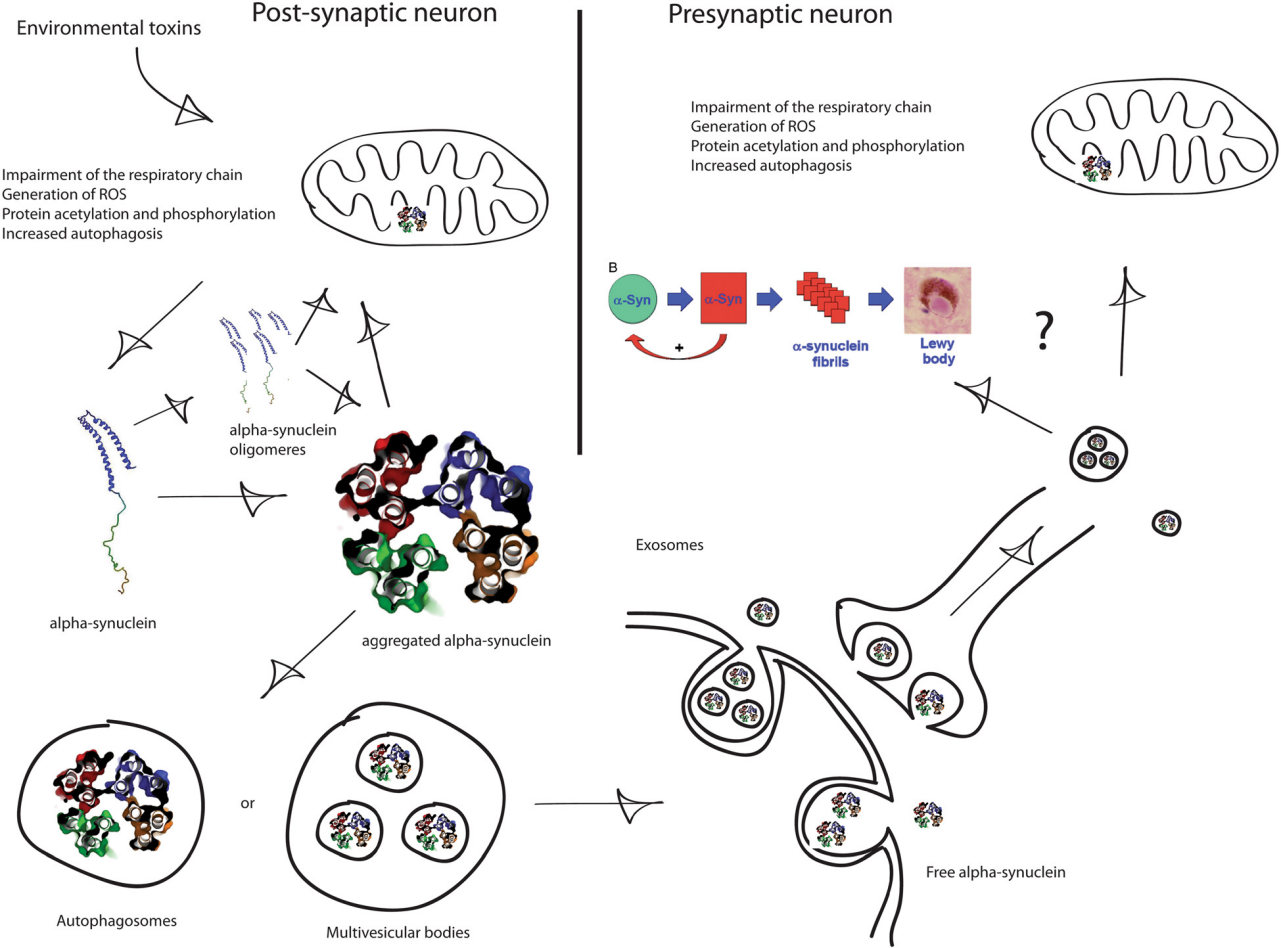
**Possible pathophysiological mechanism implicated in PD**-**like pathology progression.** Environmental toxins cause Complex I inhibition that in return increases ROS production inducing modification and impairing lysosomic/autophagic activity. This results in alpha-synuclein oligomerization and aggregation. Oligomerized or aggregated alpha-synuclein can i) interact with mitochondria inhibiting the mitochondrial respiratory system thereby multiplying the effect of the toxin or ii) be transported into autophagosomes and secreted to the extracellular environment inside or outside exosomes. Secreted alpha-synuclein is can be up-taken by presynaptic neurons and retrogradelly transported to the soma where it accumulates. The most important question here is. Does alpha-synuclein exert any effect on the presynaptic neurons? If so, we believe that there are two possible mechanisms: i) as an enucleating factor modifying the local alpha-synuclein and ii) impairing presynaptic mitochondria mimicking the effect of the environmental toxins on the ENS. Both possibilities could explain the progression of PD pathology as observed in patients.

Oxidative stress leads to the production of reactive oxygen species (ROS) and has been linked to PD [126]. ROS can modify lipids and proteins (e.g. acetylation and phosphorylation) thereby altering their normal folding and degradation. Different studies have shown that inhibition of mitochondrial Complex I enhances oxidative stress hence increasing autophagy [127]. Interestingly, we have shown that rotenone induces the accumulation and release of alpha-synuclein from enteric neurons into the extracellular space [112]. Using an in vitro system mimicking the sympathetic innervation of the gut, we also showed that released alpha-synuclein can be retrogradely transported and accumulated in the soma of the host neurons. All this is summarized in Figure 2.

This could also explain the results from some recent pathological studies performed on PD patients that had previously received intrastriatal embryonic cellular transplants. In these patients, grafted neurons showed alpha-synuclein inclusions similar to those observed in the surrounding neurons [128,129]. Recent in vivo and in vitro studies in mice could give some hints on the mechanism underlying this intra-neuronal transmission of the pathology. Desplats and colleagues have shown that alpha-synuclein is transported from the host to grafted neurons in mice and between cells in co-culture [130]. Using Thy-1 α-synuclein transgenic mice, they performed stereotaxic intrahippocampal injections of GFP-expressing mouse cortical neuronal stem cells. In vitro, they used differentiated SH-SY5Y neuronal cells over-expressing myc-tagged α-synuclein co-cultured with non-transfected SH-SY5Y cells. After some time, both grafted GFP cells and non-transfected SH-SY5Y showed alpha-synuclein inclusions. These were tyramide red or myc positive, thus demonstrating that they had been intracellularly transported. They seemed to have an enucleating effect. A later study performed by Alvarez-Erviti and colleagues showed that alpha-synuclein overexpression in SH-SY5Y induced lysosomal dysfunction and increased the release of exosomes containing alpha-synuclein to the media [131].

Finally, environmental toxins can also induce the release of pro-inflammatory signals. Observed in other neurological diseases like stroke, there is evidence for an increased inflammatory response in PD patients with microglial activation and inflammatory cytokine production [132-135]. The inflammation of the brain in early life caused by exposure to toxins, or environmental factors, has been suggested as a possible cause or contributor to the later development of PD [136]. The inflammatory process in such cases may involve activation of brain immune cells (microglia and astrocytes), which release inflammatory and neurotoxic factors that in turn produce neurodegeneration [44]. This concept first arose in the suggestion that infection with influenza virus in the pandemic of 1918 produced an increased risk of PD. Infection with certain microorganisms such as the soil bacterium Nocardia asteroides has been proposed as a risk factor for PD [137]. In animal experiments, exposure to the bacterial endotoxin lipopolysaccharide induced dopaminergic neurodegeneration [136,138,139]. However, it has been impossible to detect the presence of viral and bacterial DNA or other components inside the nervous system of PD patients. We have now clear evidence of an increased inflammatory response in PD patients with microglial activation and inflammatory cytokine production [132,140]. It has been proven that this process is due to i) the release of pro-inflammatory cytokines under oxidative stress [141] and ii) extracellular alpha-synuclein that can also promote by itself the appearance of an inflammatory reaction [142]. The real role and magnitude of this inflammatory response is unknown, but some authors maintain that they do play an important role in PD pathophysiology by perpetuating the process and causing further damages [143].

Therefore, it is likely that external factors trigger the appearance of the disease in these individuals. In fact, there are striking similarities in the effect of both mutations and environmental toxins that could explain this increased sensitivity and give hints on the pathophysiological process in PD.

### Gene-environment interactions

The known genetic mutations associated with PD can be grouped in three categories: alpha-synuclein mutations (*E46K*, *A30P* and *A53T*) and over-expression (mutations in *PARK*-*1*), defects in protein degradation (mutations in *PARK*-*2* and *PARK*-*5*) and increases in oxidative stress (mutations in *PARK*-*6* and *PARK*-*7*). The latter one leads to protein modifications, impairment of protein degradation and increases in alpha-synuclein release [76].

If we compare the alterations induced by PD-related mutations and the exposure to pesticides, it becomes clear that there are striking similarities. Impairment of mitochondrial function, protein modifications (phosphorylations and acetylations), alterations in protein degradation or the release of pro-inflammatory signals are common to many of these mutations. These alterations are also related to each other. Mitochondrial dysfunction can induce alterations in proteins and the release of pro-inflammatory signals as well as lysosomal impairment [144]. Altered proteins cannot be properly degraded and it is known that, at least one (i.e. alpha-synuclein) can induce mitochondrial dysfunction. It has been shown that both over-expression of alpha-synuclein and inhibition of mitochondrial respiration impair the lysosomal system and induce the release of alpha-synuclein to the extracellular matrix. Extracellular alpha-synuclein can then be up-taken by presynaptic neurons, where it accumulates and might impair mitochondrial function [145-147]. It has also been shown that extracellular alpha-synuclein induces an inflammatory reaction [148]. Finally, it is known that recruited inflammatory cells (i.e. microglia) further damage neuronal cells and induce oxidative stress through the release of superoxides and their phagocytic activity [143].

All this suggests that individuals carrying PD-related mutations might be more sensitive to environmental toxins and develop the disease at earlier stages of life.

In the last decade, there have been a number of studies aiming at analysing environmental damages on different genetic backgrounds. It has been shown that lipopolysaccharides enhance dopaminergic death in mice over-expressing human alpha-synuclein or its mutated forms and in Parkin-deficient mice [143,149,150]. The same kind of experiments using intraperitoneal injections of different neurotoxins has led to contradictory results. Paraquat, rotenone, maneb or MPTP had different effects on mice expressing human-alpha-synuclein or its mutated forms (i.e. A53T and A30P). In mice expressing A53T alpha-synuclein only a combination of maneb and paraquat, but not each of them alone, lead to an increased alpha-synuclein pathology throughout the CNS when compared to wild-type littermates [151]. Paraquat treatment on mice expressing human-alpha-synuclein or human-A53T-alpha-synuclein under a TH promoter increased alpha-synuclein pathology [152]. However, dopaminergic degradation was observed only in wild-type mice. This same treatment on DJ-1 mice showed that the dopaminergic neurons of these mice have an increased sensitivity to paraquat [153]. Similarly, LRRK-2 knock-out mice were as sensitive to neurotoxins as their wild-type littermates. Remarkably, it seems that, while increasing alpha-synuclein levels and the presence of alpha-synuclein pathology, these genetic backgrounds had either a protective or no effect on the susceptibility to environmental toxins.

Genetic studies have also shown that there are certain epigenetic modifications in the DNA, both nuclear and mitochondrial, of blood leucocytes and neurons from PD patients [154]. The majority of epigenetic alterations consist on methylations and alterations in the microRNA expression [154-156]. Some authors have tried to reverse these effects through the use of histone deacetylase inhibitors with different results (reviewed in [157]). It may well be that short exposure to pesticides can trigger permanent epigenetic modifications playing a role in the development of the disease.

### Conclusion: can we imply an environmental origin of PD?

Overall, all this body of evidence strongly suggest that environmental insults may play an important role in the appearance and progression of PD pathology. This is especially true for iPD. In these patients, the progression of the pathology may start from the ENS and OB and follows a predictable spatiotemporal pattern. Interestingly, these are the two nervous structures most exposed to environmental toxins and in vitro and in vivo studies suggest that environmental toxins acting on the ENS could initiate the pathology and trigger its progression through the release and transcellular transport of alpha-synuclein. This kind of progression mechanism could also explain the pattern observed in other neurodegenerative diseases. Supporting this hypothesis, a prion-like behaviour of an amyloid was characterized in Alzheimer’s disease in a recent study from Nussbaum and colleagues [158].

Despite this positive correlation with environmental toxins, to hypothesize that PD could be triggered only by exposure to environmental toxins would be, to say the least, naive. The onset of most diseases is due to a combination of external aggressors and individual genetic susceptibility to this aggression. This is clearly also the case for PD. The low incidence of PD suggests that differences in the individual genetic background and gene-environment interactions play an important role in the whole process. Results coming from different studies using neurotoxins in transgenic mice remain controversial. However, it may well be that the toxic models used up-to-date are not the ideal ones. Systemic injections of neurotoxins do not mimic the natural ways of exposure to these substances. The use of oral administered or inhaled neurotoxins may lead to different kind of results. We find very interesting that all neurotoxins used on different PD-related backgrounds induced an up-regulation of alpha-synuclein and an increase in LB-like inclusions. This is normally correlated to an increased exocytosis of alpha-synuclein [130,131] that, as mentioned above, has been shown to play a role in the progression of PD pathology. On the other hand, analysis of other types of genes (i.e. genes responsible for the protection against oxidative stress and genes coding for detoxifying enzymes) in different regions from those “a priori” expected (i.e. the ENS, the OB and the intestine) could reveal new mutations responsible for a higher susceptibility to the effect of environmental toxins. However, the new available data strongly suggests that the implications of these toxins in idiopathic PD are not merely testimonial. To consider PD mainly as a genetic disease subjected to minor external influence is akin to neglecting the effect of tobacco on lung cancer.

## Abbreviations

PD: Parkinson’s disease; iPD: Idiopathic Parkinson’s disease; MPTP: 1-methyl-4-phenyl-1,2,3,6-tetrahydropyridine; MPP+: 1-methyl-4-phenylpyridinium; 6-OHDA: 6-hydroxydopamine; CNS: Central nervous system; LB: Lewy bodies; LN: Lewy neurites; SN: Substantia nigra; OB: Olfactory bulb; DMV: Dorsal motor nucleus of the vagus; PNS: Peripheral nervous system; ENS: Enteric nervous system; REM: Rapid Eye Movement; ROS: Reactive oxygen species.

## Competing interests

The authors declare that they have no competing interests.

## Authors’ contributions

FP-M and HR wrote the manuscript. Both authors read and approved the final manuscript.
